# mSphere of Influence: Finding a Direction—How Do Mitochondria Know Where To Go?

**DOI:** 10.1128/mSphere.00321-19

**Published:** 2019-07-03

**Authors:** Lena Pernas

**Affiliations:** a Metabolism of Infection, Max Planck Institute for Biology of Ageing, Cologne, Germany

**Keywords:** host-pathogen interactions, intracellular parasites, metabolism, mitochondria, mitochondrial dynamics

## Abstract

Lena Pernas works in the field of metabolism of infection. In this mSphere of Influence article, she reflects on how work by Brian Cunniff (B. Cunniff, A. J. McKenzie, N. H. Heintz, and A. K. Howe, Mol Biol Cell 27:2662–2674, 2016, https://doi.org/10.1091/mbc.e16-05-0286) and Thomas Schwarz (G. Pekkurnaz, J. C. Trinidad, X. Wang, D. Kong, and T. L. Schwarz, Cell 158:54–68, 2014, https://doi.org/10.1016/j.cell.2014.06.007) helped her reframe the study of the interaction between a microbe and mitochondria.

## COMMENTARY

The coordination of mitochondrial function and distribution is crucial for organismal health; mice lacking the machinery that regulates mitochondrial positioning within a cell die postnatally (1). Yet, we understand little of how a mammalian cell decides when and where to place a mitochondrion that is estimated to be 0.1% of the volume of a cell. During the 2016 Mitochondrial Dynamics Keystone Symposium in Steamboat Springs, CO, I had the opportunity to see work presented by both Brian Cunniff (2) and Thomas Schwarz (3) that addressed this fundamental question. These groups used different *in vitro* models that exhibit unique mitochondrial positioning to identify signaling networks that coordinate mitochondrial distribution with cellular nutrient need and availability.

Although mitochondria are now recognized to regulate several cellular functions, these organelles are most known for their role as powerhouses. Thus, a logical prediction tested by Cunniff et al. (2) is that a cell positions mitochondria in regions of high energy demand, such as the leading edge of a migrating cell. Indeed, they reported the rapid and active trafficking of mitochondria into the leading edge of migrating cancer cells. Using a clever approach in which the leading edge was separated from the cell body using transwell-like inserts that allowed for protrusion of pseudopodia, they found increased ATP at the leading edge relative to the cell body. Importantly, the increased levels of ATP were dependent on mitochondrial respiration. They next asked whether the need for ATP at the leading edge regulated mitochondrial accumulation by manipulating activity of the nutrient sensor AMP-activated protein kinase (AMPK) (4). When levels of AMP are high, reflecting low cellular energy levels, AMPK is active and regulates energy-producing and -consuming processes to restore levels of ATP within the cell. Strikingly, they showed that mitochondria migrate to subcellular sites of AMPK activation. Conversely, inhibition of AMPK activity using optogenetic control of kinase function inhibited mitochondrial movement, elegantly demonstrating that subcellular distribution of nutrient levels, through AMPK activity, regulates mitochondrial movement. The mechanisms that underlie AMPK modulation of mitochondrial motility are yet unknown.

Pekkurnaz et al. (3) explored the link between mitochondrial distribution and nutrient availability in neurons that consist of a cell body and several cellular processes called neurites which can extend between 1 μm and 1 m. The uptake of glucose, the major energy source for mitochondria in neurons, is heterogeneous in neurites. How does a neuron ensure the presence of mitochondria at sites of glucose uptake for efficient ATP production? The authors first addressed this question by asking how an abundant supply of glucose affected mitochondrial motility in neurons. They observed that mitochondrial motility in neurons was significantly reduced when cells had higher levels of intracellular glucose. To dissect the mechanism underlying this phenomenon, they performed a biochemical analysis of the proteins that regulate mitochondrial motility, including Milton, which couples mitochondria with kinesin or dyneins to move mitochondria along microtubules and had been previously shown to be modified by the purported glucose sensor *O*-GlcNAc transferase (OGT). OGT performs metabolic flux-sensitive posttranslational modifications by catalyzing the addition of a single sugar moiety onto residues of cytoplasmic proteins. Investigating the link between OGT and Milton revealed that the glucose-dependent decrease in mitochondrial motility requires Milton O-GlcNAcylation. High intracellular glucose concentrations result in O-GlcNAcylation of Milton and a decrease in mitochondrial movement. Mutating GlcNacylated residues in Milton or decreasing OGT expression decreased the stationary mitochondrial pool *in vitro* and *in vivo*, mechanistically demonstrating how a shift in nutrient levels within a cell translates into altered mitochondrial motility and position.

The work from Cunniff et al. and Pekkurnaz et al. (2, 3) provides key insights into how a cell couples mitochondrial position and movement with nutrient need or availability. These findings are profound as they add a new layer to our understanding of mitochondrial dynamicity and cellular regulation of organellar function. Furthermore, these findings open several important questions regarding how cells coordinate other functions of mitochondria, such as lipid synthesis or antiviral signaling, with their subcellular distribution. Why are mitochondria specifically positioned in different cellular types or during different various cellular events? One fascinating example of this is during microbial infection. Reports from the early 1970s described the trafficking of host mitochondria to the subcellular sites of microbial vacuoles, including that of the human parasite Toxoplasma gondii (5–7). A microbiologist might argue that this represents microbial manipulation of host mitochondria, and indeed, during my doctoral studies in John Boothroyd’s lab, I identified the T. gondii effector protein mitochondrial association factor 1 (TgMAF1), which tethers host mitochondria to the parasite vacuole (8). However, mitochondria need to traffic to the parasite to be within the 15- to 20-nm tethering radius of TgMAF1. The work of Brian Cunniff and Thomas Schwarz reframed this question for me from a cell biologist point of view. Rather than seeing mitochondrial recruitment to the *Toxoplasma* vacuole as manipulation by the parasite, the question became why would the cell send mitochondria to the *Toxoplasma* vacuole? During my postdoctoral work in the lab of Luca Scorrano, a leader in mitochondrial dynamics, I discovered that host mitochondria change their dynamics during infection to compete with *Toxoplasma* for fatty acids and thus restrict the growth of the parasite (9). In my own lab, I am dissecting the cues that direct mitochondria to the parasite vacuole.

There are several peculiar examples of mitochondrial arrangements within cells, such as the mitochondria tightly arranged around the midpiece in sperm cells, or light-dependent positioning of mitochondria in plant cells. We are only beginning to dissect the molecular players and cues that direct mitochondria, or any other organelle for that matter, to subcellular locations or interacting partners. For example, nutrient starvation has been shown to specifically promote mitochondrion-lipid droplet interaction on detyrosinated microtubules to increase beta-oxidation (10). Lysosomes are spatially constrained during autophagy to facilitate lysosome-autophagosome fusion (11). Elucidating how and why cells position organelles will deepen our understanding of how a cell maintains its subcellular architecture and compartmentalizes its physiology.

## References

[1] Nguyen TT, Oh SS, Weaver D, Lewandowska A, Maxfield D, Schuler MH, Smith NK, Macfarlane J, Saunders G, Palmer CA, Debattisti V, Koshiba T, Pulst S, Feldman EL, Hajnóczky G, Shaw JM 2014 Loss of Miro1-directed mitochondrial movement results in a novel murine model for neuron disease. Proc Natl Acad Sci U S A 111:E3631–E3640. doi:10.1073/pnas.1402449111. 25136135 PMC4156725

[2] Cunniff B, McKenzie AJ, Heintz NH, Howe AK 2016 AMPK activity regulates trafficking of mitochondria to the leading edge during cell migration and matrix invasion. Mol Biol Cell 27:2662–2674. doi:10.1091/mbc.e16-05-0286. 27385336 PMC5007087

[3] Pekkurnaz G, Trinidad JC, Wang X, Kong D, Schwarz TL 2014 Glucose regulates mitochondrial motility via Milton modification by O-GlcNAc transferase. Cell 158:54–68. doi:10.1016/j.cell.2014.06.007. 24995978 PMC4224014

[4] Hardie DG, Schaffer BE, Brunet A 2016 AMPK: an energy-sensing pathway with multiple inputs and outputs. Trends Cell Biol 26:190–201. doi:10.1016/j.tcb.2015.10.013. 26616193 PMC5881568

[5] Friis RR 1972 Interaction of L cells and Chlamydia psittaci: entry of the parasite and host responses to its development. J Bacteriol 110:706–721. 4336694 10.1128/jb.110.2.706-721.1972 PMC247468

[6] Horwitz MA 1983 Formation of a novel phagosome by the Legionnaires’ disease bacterium (Legionella pneumophila) in human monocytes. J Exp Med 158:1319–1331. doi:10.1084/jem.158.4.1319. 6619736 PMC2187375

[7] Jones TC, Hirsch JG 1972 The interaction between Toxoplasma gondii and mammalian cells. II. The absence of lysosomal fusion with phagocytic vacuoles containing living parasites. J Exp Med 136:1173–1194. doi:10.1084/jem.136.5.1173. 4343243 PMC2139290

[8] Pernas L, Adomako-Ankomah Y, Shastri AJ, Ewald SE, Treeck M, Boyle JP, Boothroyd JC 2014 Toxoplasma effector MAF1 mediates recruitment of host mitochondria and impacts the host response. PLoS Biol 12:e1001845. doi:10.1371/journal.pbio.1001845. 24781109 PMC4004538

[9] Pernas L, Bean C, Boothroyd JC, Scorrano L 2018 Mitochondria restrict growth of the intracellular parasite Toxoplasma gondii by limiting its uptake of fatty acids. Cell Metab 27:886–897.e884. doi:10.1016/j.cmet.2018.02.018. 29617646

[10] Herms A, Bosch M, Reddy BJ, Schieber NL, Fajardo A, Ruperez C, Fernandez-Vidal A, Ferguson C, Rentero C, Tebar F, Enrich C, Parton RG, Gross SP, Pol A 2015 AMPK activation promotes lipid droplet dispersion on detyrosinated microtubules to increase mitochondrial fatty acid oxidation. Nat Commun 6:7176. doi:10.1038/ncomms8176. 26013497 PMC4446796

[11] Mohan N, Sorokina EM, Verdeny IV, Alvarez AS, Lakadamyali M 2019 Detyrosinated microtubules spatially constrain lysosomes facilitating lysosome-autophagosome fusion. J Cell Biol 218:632–643. doi:10.1083/jcb.201807124. 30567713 PMC6363446

